# Maternal depression during pregnancy and children's physical development

**DOI:** 10.7555/JBR.39.20250164

**Published:** 2025-05-21

**Authors:** Di Pi, Shuifang Lei, Wenjing Chang, Cong Liu, Yangqian Jiang, Yuanyan Dou, Jinghan Wang, Chang Wang, Haowen Zhang, Xin Xu, Hong Lyu, Bo Xu, Xiumei Han, Xiaoyu Liu, Kun Zhou, Tao Jiang, Jiangbo Du, Guangfu Jin, Hongxia Ma, Hongbing Shen, Zhibin Hu, Kan Ye, Yuan Lin

**Affiliations:** 1 State Key Laboratory of Reproductive Medicine, Nanjing Medical University, Nanjing, Jiangsu 211166, China; 2 Department of Maternal, Child and Adolescent Health, School of Public Health, Nanjing Medical University, Nanjing, Jiangsu 211166, China; 3 Department of Child Health Care, the Affiliated Suzhou Hospital of Nanjing Medical University, Suzhou Municipal Hospital, Gusu School, Nanjing Medical University, Suzhou, Jiangsu 215002, China; 4 Department of Epidemiology, Center for Global Health, School of Public Health, Nanjing Medical University, Nanjing, Jiangsu 211166, China; 5 State Key Laboratory of Reproductive Medicine (Suzhou Center), the Affiliated Suzhou Hospital of Nanjing Medical University, Suzhou Municipal Hospital, Gusu School, Nanjing Medical University, Suzhou, Jiangsu 215002, China; 6 Department of Biostatistics, School of Public Health, Nanjing Medical University, Nanjing, Jiangsu 211166, China

**Keywords:** prenatal distress, children, overweight and obesity, cohort study

## Abstract

Prenatal maternal psychological distress, particularly depression, has been increasingly recognized as a factor that influences fetal growth; however, its impact on early childhood development remains less well understood. The present study investigated the association between prenatal depression and children's growth trajectories, as well as the odds of overweight and obesity from 1 to 36 months, while also accounting for maternal anxiety and stress. We analyzed data from 4710 mother-child dyads in the Jiangsu Birth Cohort, assessing maternal psychological distress across trimesters and categorizing participants into groups with mild, moderate, and severe depressive symptomatology. Children's weight-for-length z-scores (WLZ) were used to assess overweight/obesity prevalence, and growth patterns were identified through trajectory models. The results from the generalized estimating equations analysis showed that greater depressive symptomatology during pregnancy was associated with a 28% to 41% increase in the odds of childhood overweight/obesity across all three trimesters, compared with mild depressive symptomatology. We identified five distinct WLZ growth trajectory patterns, and found that mothers with greater depressive symptomatology were 39%–47% more likely to have children who followed a very-high-stable growth trajectory, compared with mothers with mild depressive symptomatology. These findings highlight the significant impact of prenatal depression on adverse growth patterns and childhood overweight/obesity, underscoring the need for early intervention.

## Introduction

Childhood overweight and obesity have become a growing global public health concern. Reports indicate that obesity rates increased by 1.5-fold between 2012 and 2023 compared with those in the period from 2000 to 2011, and that the prevalence of overweight and obesity in children and adolescents reached approximately 8.5% during 2000–2023^[1]^. In China, the trend mirrors global patterns, particularly among preschool-aged children. In 2019, an estimated 6.8% of preschool children in China were classified as overweight, while 3.6% were classified as obese^[2]^. These early weight issues often persist, as overweight children are five times more likely to remain obese in adolescence and adulthood compared with their non-obese peers^[3]^. Moreover, childhood obesity is associated with an increased risk of comorbidities such as cardiovascular diseases and type 2 diabetes^[4–5]^.

Psychological distress during pregnancy, even if it does not reach the severity of a mental disorder, can have adverse effects on both the mother and the child^[6]^. According to the Developmental Origins of Health and Disease (DOHaD) hypothesis^[7]^, maternal exposure to adverse psychological states during pregnancy, along with associated physiological and lifestyle changes, can directly or indirectly influence offspring's health and development^[8–9]^. A substantial body of research has shown that maternal psychological factors significantly influence fetal development^[10–11]^. However, evidence regarding the impact of maternal psychological factors on children's postnatal physical growth remains limited. One Canadian study reported that elevated maternal stress during pregnancy was associated with an increased body mass index (BMI) in children and a heightened risk of central obesity^[12]^. Similarly, a cohort study reported that perinatal anxiety was moderately associated with increased BMI in children by age two, though no significant association was observed between maternal depression and children's BMI^[13]^. Notably, evidence on the associations between maternal prenatal distress and physical development in children remains inconsistent. Some studies have suggested that maternal anxiety during the third trimester was associated with lower BMI in children aged 48 to 72 months^[14]^. These conflicting results highlight the need for further investigation into the relationship between maternal psychological distress during pregnancy and physical development in children. Additionally, previous studies have primarily relied on conventional analytical methods, such as linear mixed-effects models and generalized estimating equations (GEEs), to assess postnatal physical growth^[12,15]^. A significant gap remains in investigations into the association between maternal psychological distress and children's physical growth trajectories, which can offer a more comprehensive description of the impact of prenatal exposure on children's dynamic growth processes^[16]^. To fill this knowledge gap, the present study used data from a prospective birth cohort to investigate the associations between maternal prenatal depression and children's risk of overweight/obesity and growth trajectories from 1 to 36 months of age, while also exploring the additional effects of anxiety and stress.

## Subjects and methods

### Study design and population

The Jiangsu Birth Cohort (JBC) study is an ongoing multi-center prospective cohort study in Jiangsu, China, enrolling families either seeking assisted reproductive technology treatment or conceiving naturally^[17]^. The JBC study recruited participants at three major hospitals in Jiangsu, namely the Women's Hospital Affiliated to Nanjing Medical University (Nanjing), Suzhou Hospital Affiliated to Nanjing Medical University (Suzhou), and Changzhou Maternity and Child Health Care Hospital Affiliated to Nanjing Medical University (Changzhou). Between August 2016 and June 2020, 9016 pregnant women were recruited. After excluding women with multiple gestations, preterm births, neonatal/infant mortality, and major birth defects, 7732 mother-child dyads remained. In the present study, we further excluded 819 women without psychological assessments during pregnancy, 906 children lost to follow-up, and 1297 children with fewer than three weight and length/height measurements across nine follow-up points (1, 3, 6, 8, 12, 18, 24, 30, and 36 months). A total of 4710 mother-child dyads were included in the final analysis (***Supplementary Fig. 1***). The study was approved by the Institutional Review Board of Nanjing Medical University [NJMUIRB(2014)248], and all participants provided written informed consent.

### Exposures

In the present study, participants were required to complete at least one psychological assessment (depression, anxiety, or stress) during any of the three trimesters. Maternal psychological distress during pregnancy was assessed using three validated self-reported questionnaires at the first (8–14 weeks), second (22–26 weeks), and third trimesters (30–34 weeks). The Perceived Stress Scale (PSS-10)^[18]^ is a 10-item questionnaire used to assess an individual's perception of general stress and coping over the past month. The Self-Rating Anxiety Scale (SAS)^[19]^ is a 20-item scale designed to evaluate anxiety levels. The Center for Epidemiologic Studies Depression Scale (CES-D)^[20]^ is a 20-item tool used to assess depressive symptoms over the past seven days and is widely used in epidemiological research. Higher scores on all scales indicate greater distress. The original scoring standards for these scales were developed based on the general population and may not fully align with the characteristics of pregnant women in the present study. Thus, participants were categorized into mild, moderate, and severe symptomatology based on the distribution of their scores. The correspondence between tertile-based categorization and standard scoring thresholds is provided in ***Supplementary Table 1***.

### Outcomes

Children's weight and length were measured by trained healthcare professionals at nine scheduled follow-up points from 1 to 36 months of age (1, 3, 6, 8, 12, 18, 24, 30, and 36 months). We calculated weight-for-length z-scores (WLZ) using the World Health Organization reference standards for children under five years old^[21]^. Children with WLZ > 2 were classified as overweight, and those with WLZ > 3 were classified as obese. The exact age of each child at the time of measurement was calculated by subtracting the date of birth from the measurement date, ensuring accurate anthropometric assessments.

### Covariates

In our analysis, a Directed Acyclic Graph (DAG) was used to select relevant covariates (***Supplementary Fig. 2***). Maternal and child data were collected during pregnancy or follow-up visits through self-reported questionnaires or the hospitals' electronic medical records. The covariates identified by the DAG that required adjustment include annual household income, pre-pregnancy BMI, age at recruitment, education level, place of residence, and parity. Maternal age refers to the age of the mother at recruitment, and pre-pregnancy BMI was calculated by dividing pre-pregnancy weight by the height square (kg/m²).

### Statistical analysis

The GEE approach was used to assess the associations between prenatal maternal depression and WLZ, as well as the incidence of overweight/obesity in children aged 1 to 36 months. GEE is a statistical method for analyzing longitudinal data with repeated measures, accounting for correlations between repeated observations within the same individual, thereby providing population-averaged effect estimates^[22]^. We employed an autoregressive correlation structure to account for correlations between repeated measurements for each child. Logistic regression models were used to examine the relationship between prenatal maternal depression and overweight/obesity at different time points. The significance of trends was tested by including the median psychological score within each tertile as a continuous variable in the regression models. To assess the trends in maternal psychological health during pregnancy and children's physical development, we performed the Jonckheere-Terpstra test for trend and a quadratic term trend test using a generalized linear model. Model 1 was adjusted for annual household income, pre-pregnancy BMI, age at recruitment, education level, place of residence, and parity. Model 2 was adjusted based on Model 1, with the addition of breastfeeding duration. Missing covariate data were handled *via* multiple imputation, generating 50 datasets using the "mice" package.

GBTM (group-based trajectory modeling) was used to identify the physical growth trajectories of WLZ. By employing a polynomial function, GBTM established the relationship between time and growth, grouping individuals with similar developmental trends into the same subgroup. To determine the optimal number of trajectory subgroups, we started with one trajectory and the lowest polynomial order, progressively refining the model for the best fit. The selection criteria included: (1) average group posterior probability (AvePP) > 0.7; (2) odds of correct classification > 5; (3) a practical and efficient model (with a lower Bayesian Information Criterion [BIC] value); and (4) a subgroup proportion (Prop) > 0.05 for each group.

Stratified analyses were conducted by mode of conception, infant sex, and pre-pregnancy BMI, with interaction tests performed. Several sensitivity analyses were also conducted. First, we excluded individuals classified as large for gestational age (LGA) or small for gestational age (SGA) according to the INTERGROWTH-21^st^ standards 30 to assess whether prenatal maternal depression affects physical development in children with birth weights between the 10th and 90th percentiles. Second, we repeated the analysis by excluding individuals with gestational diabetes or gestational hypertension. Finally, we reanalyzed the data after excluding individuals with missing covariate data.

All analyses were conducted using R software (version 4.3.2, R Foundation for Statistical Computing, Vienna, Austria). *P*-values less than 0.05 were considered statistically significant.

## Results

### Characteristics of participants

The characteristics of the cohort participants are presented in ***Table 1***. The analysis included 4710 mother-singleton dyads, with 12.63% of mothers aged 35 years or older, and 76.24% being first-time mothers. Over half of the infants were males (*n* = 2480 infants, 52.65%). The baseline characteristic comparisons are presented in the supplementary tables. ***Supplementary Table 2*** shows the baseline characteristics stratified by tertiles of maternal depressive symptoms for each trimester, while ***Supplementary Table 3*** compares the baseline characteristics between participants included in the final analysis and those excluded. Depression scores showed a statistically significant decreasing trend across pregnancy (*P* < 0.001) (***Supplementary Table 4***). The prevalence of overweight/obesity in children showed a significant non-linear trend (*P* < 0.001). Specifically, the prevalence increased to 9.75% at six months, then gradually declined to 3.51% by 24 months (***Supplementary Table 5***).

**Table 1:** Baseline characteristics of 4710 mothers and their children

Characteristics	Study population (*n* = 4710)
Mothers	
Place of residence [*n* (%)]	
Rural	534 (11.34)
Urban/Suburban	4176 (88.66)
Annual household income [10000 CNY, *n* (%)]	
< 5	164 (3.49)
[5, 10)	1049 (22.32)
[10, 20)	2030 (43.19)
≥ 20	1457 (31.00)
Age at recruitment [*n* (%)]	
< 35	4115 (87.37)
≥ 35	595 (12.63)
Pre-pregnancy BMI [kg/m^2^, *n* (%)]	
< 18.5	557 (11.83)
[18.5, 24.0)	3224 (68.49)
[24.0, 28.0)	712 (15.13)
≥ 28.0	214 (4.55)
Education level [years, *n* (%)]	
< 12	808 (17.16)
≥ 12	3900 (82.84)
Parity [*n* (%)]	
Nulliparous	3588 (76.24)
Multiparous	1118 (23.76)
Smoking during pregnancy [*n* (%)]	19 (0.40)
Drinking during pregnancy [*n* (%)]	43 (0.91)
Diabetes during pregnancy [*n* (%)]	
No	3387 (72.29)
GDM	1219 (26.02)
DM	79 (1.69)
GH [*n* (%)]	249 (5.33)
Mode of conception [*n* (%)]	
ART	1570 (33.33)
Spontaneous	3140 (66.67)
Children	
Gestational weeks at delivery (weeks, mean ± SD)	39.58 ± 1.00
Infant sex [*n* (%)]	
Male	2480 (52.65)
Female	2230 (47.35)
Birth weight (g, mean ± SD)	3409.90 ± 403.82
SGA [*n* (%)]	167 (3.55)
LGA [*n* (%)]	666 (14.15)
Breastfeeding duration [months, *n* (%)]	
< 6	798 (16.99)
≥ 6	3900 (83.01)
Missing (*n*): Annual household income (10), parity (4), pre-pregnancy BMI (3), education level (2), smoking during pregnancy (4), drinking during pregnancy (3), diabetes during pregnancy (25), GH (35), LGA (2), SGA (2), breastfeeding duration (12).Abbreviations: ART, assisted reproductive technology; BMI, body mass index; DM, diabetes mellitus; GDM, gestational diabetes mellitus; GH, gestational hypertension; LGA, large for gestational age; SD, standard deviation; SGA, small for gestational age.	

### Maternal depression and overweight/obesity in children

The results of the GEE analysis revealed that greater depressive symptomatology during pregnancy was associated with an increased likelihood of overweight/obesity in children aged 1 to 36 months. This relationship displayed a significant linear trend across trimesters (*P* for trend < 0.05). Specifically, compared with mild depressive symptomatology, more depressive symptomatology during the first trimester was associated with 32% higher odds of childhood overweight/obesity (odds ratio [OR] = 1.32, 95% confidence interval [CI], 1.06–1.63); during the second trimester, with 41% higher odds (OR = 1.41, 95% CI, 1.12–1.78); and during the third trimester, with 28% higher odds (OR = 1.28, 95% CI, 1.01–1.62) in the crude model. After adjusting for the covariates, including annual household income, pre-pregnancy BMI, age at recruitment, education level, place of residence, parity, and breastfeeding duration, the significant associations remained. Women exposed to more depressive symptomatology during the first, second, and third trimesters were associated with 29% to 40% higher odds of children with overweight/obesity in their early childhood compared with those with mild depressive symptomatology (first trimester adjusted OR [aOR] = 1.36, 95% CI, 1.10–1.69; second trimester aOR = 1.40, 95% CI, 1.10–1.77; third trimester aOR = 1.29, 95% CI, 1.01–1.63). However, those with moderate depressive symptomatology did not show significantly higher odds of overweight/obesity in children compared with those with mild depressive symptomatology (***Table 2***). No associations were found between prenatal stress and anxiety and childhood overweight/obesity (***Supplementary Table 6***).

**Table 2:** Associations between prenatal depression and children's overweight/obesity from 1 to 36 months

Groups	Crude			Model 1			Model 2	
	OR (95% CI)	*P*-value		OR (95% CI)	*P*-value		OR (95% CI)	*P*-value
First trimester depression score								
Tertile 1	Reference			Reference			Reference	
Tertile 2	0.99 (0.80, 1.23)	0.924		0.99 (0.80, 1.22)	0.919		0.99 (0.80, 1.22)	0.904
Tertile 3	1.32 (1.06, 1.63)	0.012		1.36 (1.10, 1.69)	0.005		1.36 (1.10, 1.69)	0.005
*P* for trend		0.010			0.004			0.004
Second trimester depression score								
Tertile 1	Reference			Reference			Reference	
Tertile 2	1.11 (0.87, 1.41)	0.417		1.10 (0.86, 1.40)	0.458		1.09 (0.85, 1.39)	0.494
Tertile 3	1.41 (1.12, 1.78)	0.004		1.42 (1.12, 1.79)	0.004		1.40 (1.10, 1.77)	0.005
*P* for trend		0.003			0.003			0.004
Third trimester depression score								
Tertile 1	Reference			Reference			Reference	
Tertile 2	1.01 (0.78, 1.31)	0.927		1.01 (0.79, 1.31)	0.915		1.00 (0.78, 1.30)	0.975
Tertile 3	1.28 (1.01, 1.62)	0.043		1.31 (1.03, 1.66)	0.028		1.29 (1.01, 1.63)	0.039
*P* for trend		0.035			0.022			0.031
Model 1 was adjusted for annual household income, pre-pregnancy BMI, age at recruitment, education level, place of residence, and parity. Model 2 was adjusted based on Model 1, with the addition of breastfeeding duration. The associations were estimated using the generalized estimating equation (GEE) models. The *P* for trend was calculated by including the median maternal depression score within each tertile as a continuous variable in the regression model to assess the significance of trends across ordered groups.Abbreviations: BMI, body mass index; CI, confidence interval; OR, odds ratio.								

**Figure 1:**
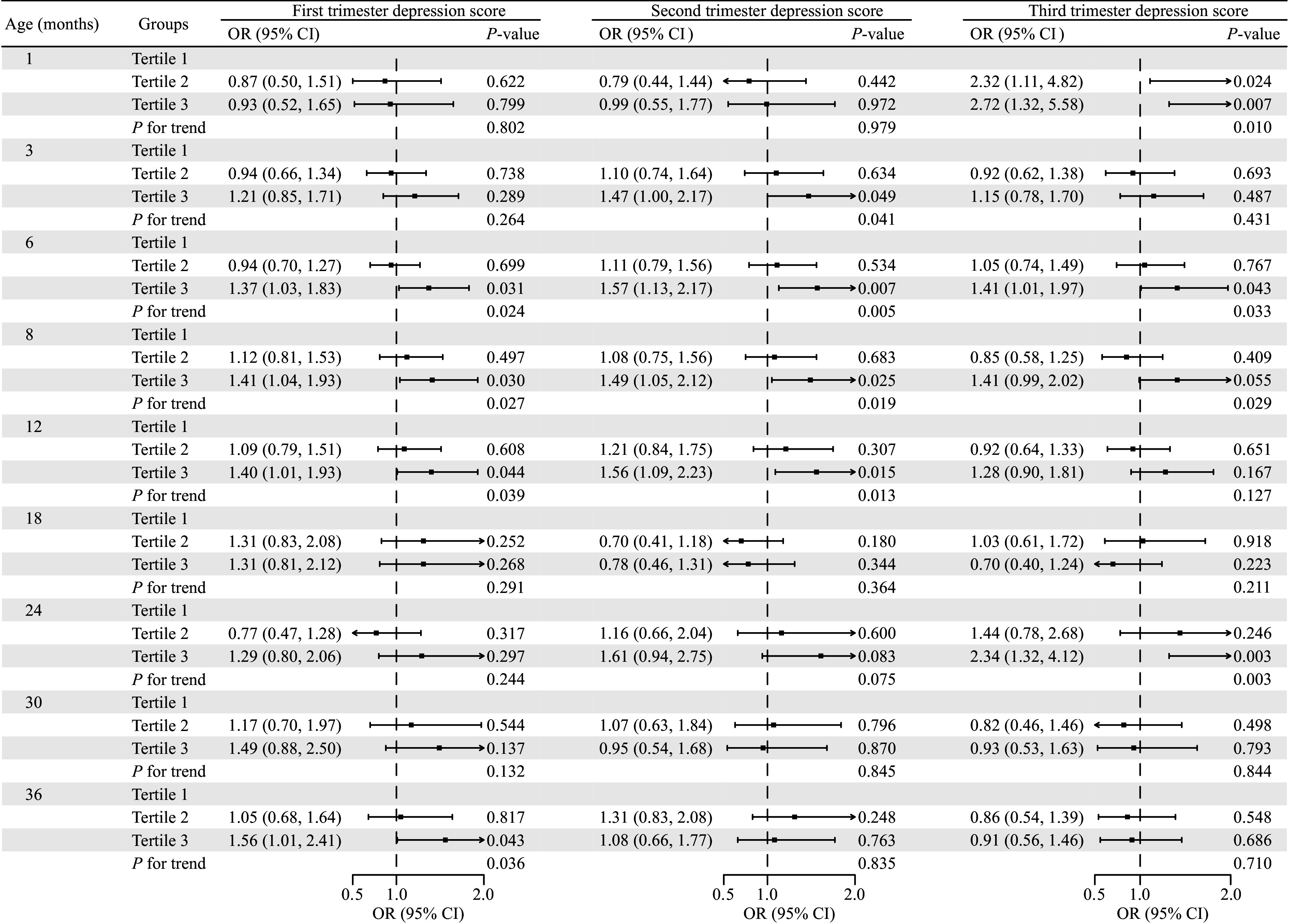
The associations between maternal depression in each trimester and overweight/obesity in children at different ages. The associations were estimated using logistic regression models. The *P* for trend was calculated by including the median maternal depression score within each tertile as a continuous variable in the regression model to assess the significance of trends across ordered groups. The model was adjusted for annual household income, pre-pregnancy BMI, age at recruitment, education level, residence place, parity, and breastfeeding duration. Abbreviations: BMI, body mass index; CI, confidence interval; OR, odds ratio.

Multi-timepoint analysis revealed that more depressive symptomatology during pregnancy was associated with higher odds of children with overweight/obesity at most time points between 1 and 36 months, with variations observed across different gestational periods. Specifically, more depressive symptomatology during the third trimester was significantly associated with higher odds of overweight/obesity at one month of age (aOR = 2.72, 95% CI, 1.32–5.58), whereas no significant associations were observed for first- and second-trimester depression at this time point. More depressive symptomatology during the first, second, and third trimesters was consistently associated with higher odds of overweight/obesity in children from 3 to 12 months, with relative increases ranging from 15% to 57%. Beyond 12 months, significant associations were observed primarily for first-trimester exposure (***Fig. 1***).

Using GEE models, we observed that greater depressive symptomatology during the first and second trimesters was significantly associated with a more rapid increase in WLZ from 1 to 36 months of age (first trimester: a*β* = 0.09, 95% CI, 0.02–0.15; second trimester: a*β* = 0.07, 95% CI, 0.01–0.14). Although the third trimester results were not statistically significant, the direction and magnitude of the associations were similar to those observed for the first two trimesters (***Supplementary Table 7***).

### Maternal depression and growth patterns in children

GBTM identified five distinct WLZ trajectory groups: very-high-stable, high-stable, moderate-stable, low-rising, and low-stable (***Supplementary Fig. 3***). A detailed description of the BIC values and posterior probabilities used for model selection and fit is provided in ***Supplementary Table 8***. The moderate-stable trajectory group represented the majority of the study population, with an average z-score near zero during the first 36 months of life, serving as the reference group. The distribution of these five trajectory groups at different ages is also displayed (***Supplementary Table 9***).

**Figure 2:**
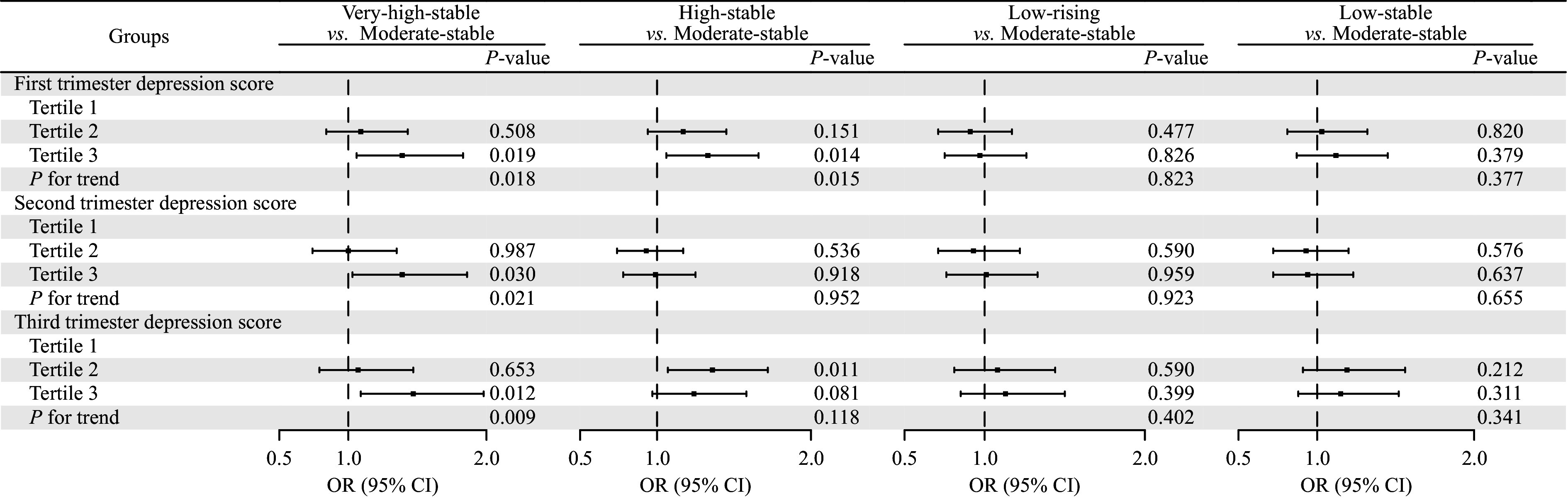
Associations between maternal depression in each trimester and growth trajectories in children from 1 to 36 months. The associations were estimated using multinomial logistic regression models. The *P* for trend was calculated by including the median maternal depression score within each tertile as a continuous variable in the regression model to assess the significance of trends across ordered groups. The model was adjusted for annual household income, pre-pregnancy BMI, age at recruitment, education level, residence place, parity, and breastfeeding duration. Abbreviations: BMI, body mass index; CI, confidence interval; OR, odds ratio.

More depressive symptomatology was associated with a higher likelihood of children falling into the very-high-stable trajectory group than into the moderate-stable group, showing a significant linear trend across trimesters (*P* for trend < 0.05, see ***Fig. 2***). Specifically, after adjusting for covariates including annual household income, pre-pregnancy BMI, age at recruitment, education level, place of residence, parity, and breastfeeding duration, compared with mild depressive symptomatology, more depressive symptomatology was associated with higher odds of adverse child growth outcomes: more depressive symptomatology during the first trimester was linked to a 39% higher odds (aOR = 1.39, 95% CI, 1.06–1.83), a 39% higher odds during the second trimester (aOR = 1.39, 95% CI, 1.03–1.86), and a 47% higher odds during the third trimester (aOR = 1.47, 95% CI, 1.09–1.98). Additionally, compared with mild depressive symptomatology, more depressive symptomatology during the first trimester was associated with a 33% higher odds of children falling into the high-stable trajectory group than into the moderate-stable group (aOR = 1.33, 95% CI, 1.06–1.66) (***Supplementary Table 10***).

Stratified analyses revealed that infant sex, mode of conception, and pre-pregnancy BMI did not significantly modify the association between maternal depression and child physical growth. Sensitivity analyses yielded consistent results with the main analysis. After excluding LGA and SGA infants, the association remained unchanged. Excluding participants with gestational diabetes or gestational hypertension also yielded consistent results, although some 95% CIs included 1. Furthermore, the results remained stable even after excluding participants with incomplete covariate data (***Supplementary Tables 11***–***14***).

## Discussion

The present study found that greater depressive symptomatology during pregnancy was significantly associated with higher odds of childhood overweight/obesity and a higher likelihood of children belonging to the very-high-stable growth trajectory group. Although prenatal stress and anxiety showed similar trends, they did not reach statistical significance.

Depression is one of the most common psychological issues during pregnancy and is often accompanied by increased psychological stress. Roddy Mitchell *et al*^[23]^ reported a pooled prevalence of perinatal depression of 24.7%. Further analysis of regional data revealed that the prevalence of perinatal depression was relatively lower in East Asia and the Pacific (21.4%) and significantly higher in the Middle East and North Africa (31.5%). These variations may be influenced by geographical, socioeconomic, and cultural differences. The same study also identified several key risk factors for antenatal depression, including exposure to intimate partner violence, HIV infection, adolescent pregnancy, and residence in conflict-affected settings. The present study systematically evaluated the association between maternal depression during pregnancy and offspring physical development from 1 to 36 months of age, while also accounting for the potential effects of anxiety and stress. Previous studies on this topic have yielded conflicting results. For instance, a cohort study from the UK found no association between prenatal depression and children's BMI^[13]^, while a German cohort study observed a link between prenatal depression and increased BMI in children^[24]^. Our study aligns with the latter, further suggesting that prenatal depression may influence physical development in children. Existing studies suggest that prenatal psychological stress is associated with children's BMI and the odds of overweight/obesity^[25]^. However, our findings did not show a significant association between prenatal stress and the odds of overweight/obesity. Similarly, although a UK study reported a link between maternal anxiety symptoms and increased BMI in children^[13]^, our findings also did not show a significant association between prenatal anxiety and the odds of overweight/obesity. Overall, our findings underscore that elevated maternal psychological distress during pregnancy, particularly depression, has a substantial impact on childhood obesity odds. Methodological variations across studies, such as differences in measurement tools, may contribute to discrepancies and should be considered when interpreting results.

The impact of depression on children's physical development appears to vary as children grow up. For instance, a study conducted in Quebec found that the older the child, the stronger the positive correlation between maternal objective stress during pregnancy and the child's body composition^[12]^. In our study, this effect remained relatively stable before 12 months of age. After 12 months, the significant association was mainly concentrated in the first-trimester exposure. Similarly, a cohort study conducted in Australia^[26]^ found that psychological distress during the first trimester, compared with that experienced in the third trimester, had a more consistent influence on accelerated growth and obesity odds in children aged 3 to 14 years. In contrast, findings from a mouse model study indicated that offspring exposed to stress during the second and third trimesters exhibited more pronounced long-term weight gain^[27]^. Currently, research on the exposure windows of prenatal psychological distress and its effects on offspring's physical development remains limited. Further studies are needed to explore this relationship in more detail.

In the present study, we further explored the association between maternal depression during pregnancy and children's growth trajectories. To date, limited research has examined these dynamics. A study from the Ma'anshan birth cohort in China investigated associations between prenatal anxiety and growth patterns in children aged 48 to 72 months, reporting that the third-trimester anxiety increased the likelihood of children being in an extremely low BMI trajectory group^[14]^. Our study constructed five WLZ trajectories, similar to those reported in other Chinese birth cohorts^[28–29]^. Our analysis revealed that higher maternal depressive scores during pregnancy were associated with an increased likelihood of children belonging to the high-stable growth trajectory group, further supporting the conclusion that elevated prenatal depression increases the odds of childhood overweight and obesity.

Maternal depression during pregnancy may influence children's growth and development through multiple biological and behavioral mechanisms. First, depression can disrupt maternal prenatal health behaviors, such as increasing the likelihood of smoking or adopting unhealthy dietary patterns, which may negatively impact fetal growth^[30]^. Second, antenatal depression may abnormally activate the hypothalamic-pituitary-adrenal (HPA) axis, altering the intrauterine endocrine environment and impairing fetal physiological and developmental processes^[31]^. Additionally, maternal depression may affect immune function and fetal brain development, potentially contributing to adverse long-term health outcomes in offspring^[32–33]^. Emerging evidence also suggests that psychological stress and emotional distress during pregnancy can alter DNA methylation patterns in the placenta or fetus, thereby affecting the expression of genes critical for development and increasing the risk of later-life health issues such as obesity and metabolic disorders^[34]^. Overall, maternal mental health during pregnancy not only affects maternal physiology but also directly influences fetal development, potentially laying the foundation for future health issues such as childhood obesity. Given these mechanisms, early identification and intervention for maternal psychological distress during pregnancy are crucial. Regular mental health screenings, psychological counseling, and stress reduction programs such as cognitive behavioral therapy and mindfulness-based approaches are effective interventions^[35]^. These measures not only help alleviate maternal psychological distress but also improve the growth trajectories of children, thereby reducing the risk of adverse health outcomes.

The present study has several strengths. First, repeated measurements of children from 1 to 36 months and maternal psychological assessments at three time points during pregnancy enhanced the reliability of the results. Second, the study explored both the associations between maternal psychological distress and children's physical development, as well as their growth trajectories, providing greater depth to the analysis. Lastly, the study controlled for potential confounders and validated the results through sensitivity analyses.

However, the present study has several limitations. First, maternal psychological status was assessed using self-reported questionnaires, which may be subject to recall bias and differences in interpretation, potentially leading to underreporting or overreporting. Second, the study may be influenced by unmeasured confounding factors, as data on maternal treatment and medication use, nutritional status, and lifestyle factors were not considered, which could introduce bias into the findings. Third, there was a certain degree of missing data and potential selection bias. Fourth, preterm infants were excluded from the analysis. Given that preterm birth is associated with higher levels of maternal psychological distress and that preterm infants may differ in both physiological and psychological characteristics, excluding this group may have led to an underestimation of the impact of maternal distress on offspring development, as well as limited the generalizability of the findings. Finally, the absence of biological markers for anxiety and depression (such as cortisol or other stress hormones) constrained further exploration of the underlying physiological mechanisms.

### Conclusions

The present study shows that prenatal depression increases the odds of childhood overweight/obesity and affects children's growth trajectories. It highlights the importance of routine mental health screenings during pregnancy, with an emphasis on depressive symptoms. Early identification and intervention for prenatal depression can help reduce its negative impact on maternal and infant health. Future research should explore how prenatal psychological factors influence children's physical development through physiological mechanisms.

## Additional information

The online version contains supplementary material available at http://www.jbr-pub.org.cn/article/doi/10.7555/JBR.39.20250164?pageType=en.
