# Supplementary material for: Maternal depression during pregnancy and children's physical development
Source: J Biomed Res. 2025 May 21;40(1):1–10. doi: 10.7555/JBR.39.20250164 (PMC12794177; doi:10.7555/JBR.39.20250164)
Supplement: Supplementary file 1 — The online version contains supplementary material available at http://www.jbr-pub.org.cn/article/doi/10.7555/JBR.39.20250164?pageType=en. [file jbr-40-1-1-S1.pdf]

Available online at www.jbr-pub.org.cn

Open Access at PubMed Central

Journal of Biomedical Research, 2026 40(1): 1–10

Supplementary Data

JBR

Maternal depression during pregnancy and children's physical development

Di Pi[1,2,△], Shuifang Lei[1,2,△], Wenjing Chang[3,△], Cong Liu[1,4], Yangqian Jiang[1,2], Yuanyan Dou[1,4], Jinghan Wang[1,2], Chang Wang[1,2], Haowen Zhang[1,2,5], Xin Xu[1,2], Hong Lyu[1,2], Bo Xu[1,4], Xiumei Han[1,4], Xiaoyu Liu[1,4], Kun Zhou[1,4], Tao Jiang[1,6], Jiangbo Du[1,4,5], Guangfu Jin[1,4,5], Hongxia Ma[1,4,5], Hongbing Shen[1,4,5], Zhibin Hu[1,4,5], Kan Ye[3,✉], Yuan Lin[1,2,5,✉]

[1]*State Key Laboratory of Reproductive Medicine, Nanjing Medical University, Nanjing, Jiangsu 211166, China;*
[2]*Department of Maternal, Child and Adolescent Health, School of Public Health, Nanjing Medical University, Nanjing, Jiangsu 211166, China;*
[3]*Department of Child Health Care, the Affiliated Suzhou Hospital of Nanjing Medical University, Suzhou Municipal Hospital, Gusu School, Nanjing Medical University, Suzhou, Jiangsu 215002, China;*
[4]*Department of Epidemiology, Center for Global Health, School of Public Health, Nanjing Medical University, Nanjing, Jiangsu 211166, China;*
[5]*State Key Laboratory of Reproductive Medicine (Suzhou Center), the Affiliated Suzhou Hospital of Nanjing Medical University, Suzhou Municipal Hospital, Gusu School, Nanjing Medical University, Suzhou, Jiangsu 215002, China;*
[6]*Department of Biostatistics, School of Public Health, Nanjing Medical University, Nanjing, Jiangsu 211166, China.*

Supplementary Table 1 **Tertile and standard scoring correspondence for maternal psychological scales**

Trimesters	Groups	SAS score range	*N*	Positive [*n* (%)]	CES-D score range	*N*	Positive [*n* (%)]	PSS-10 score range	*N*	Positive [*n* (%)]
First trimester	Tertile 1	< 35	1 263	0 (0)	< 8	1 194	0 (0)	< 10	1 188	0 (0)
	Tertile 2	35–41	1 140	0 (0)	8–16	1 275	123 (9.6)	10–15	1 219	236 (19.4)
	Tertile 3	> 41	1 154	328 (28.4)	> 16	1 088	1 088 (100.0)	> 15	1 150	1 150 (100.0)
Second trimester	Tertile 1	< 32	1 170	0 (0)	< 4	1 113	0 (0)	< 7	1 123	0 (0)
	Tertile 2	32–38	1 034	0 (0)	4–11	1 049	0 (0)	7–13	1 116	0 (0)
	Tertile 3	> 38	938	139 (14.8)	> 11	980	587 (59.9)	> 13	903	738 (81.7)
Third trimester	Tertile 1	< 32	1 004	0 (0)	< 4	1 025	0 (0)	< 6	989	0 (0)
	Tertile 2	32–40	1 093	0 (0)	4–11	967	0 (0)	6–12	1 017	0 (0)
	Tertile 3	> 40	845	173 (20.5)	> 11	950	565 (59.5)	> 12	936	685 (73.2)

The cutoff scores for case definition were defined as SAS > 50; CES-D > 15; PSS-10 > 14.
Abbreviations: CES-D, Center for Epidemiologic Studies Depression Scale; PSS, Perceived Stress Scale; SAS, Self-Rating Anxiety Scale.

△These authors contributed equally to this work.

✉Corresponding authors: Yuan Lin, Department of Maternal, Child and Adolescent Health, Center for Global Health, School of Public Health, Nanjing Medical University, Nanjing, Jiangsu 211166, China. E-mail: yuanlin@njmu.edu.cn; Kan Ye, Department of Child Health Care, the Affiliated Suzhou Hospital of Nanjing Medical University, Suzhou Municipal Hospital, Gusu School, Nanjing Medical University, Suzhou, Jiangsu 215002, China. E-mail: yekan001@163.com.

Received: 16 April 2025; Revised: 11 May 2025; Accepted: 18 May 2025; Published online: 21 May 2025

CLC number: R714.25, Document code: A

The authors reported no conflict of interests.

This is an open access article under the Creative Commons Attribution (CC BY 4.0) license, which permits others to distribute, remix, adapt and build upon this work, for commercial use, provided the original work is properly cited.

© 2026 by Journal of Biomedical Research.

https://doi.org/10.7555/JBR.39.20250164

Supplementary Table 2 **Baseline characteristics by maternal depressive symptom tertiles during each trimester**

Characteristics	First trimester depression				Second trimester depression				Third trimester depression			
	Tertile 1 (n = 1 194)	Tertile 2 (n = 1 275)	Tertile 3 (n = 1 088)	P-value	Tertile 1 (n = 1 113)	Tertile 2 (n = 1 049)	Tertile 3 (n = 980)	P-value	Tertile 1 (n = 1 025)	Tertile 2 (n = 967)	Tertile 3 (n = 950)	P-value
Mothers												
Place of residence [n (%)]				0.054				0.229				0.096
Rural	183 (15.33)	173 (13.57)	129 (11.86)		59 (5.30)	42 (4.00)	54 (5.51)		71 (6.93)	45 (4.65)	57 (6.00)	
Urban/Suburban	1 011 (84.67)	1 102 (86.43)	959 (88.14)		1 054 (94.70)	1 007 (96.00)	926 (94.49)		954 (93.07)	922 (95.35)	893 (94.00)	
Annual household income [10 000 CNY, n (%)]				0.234				0.011				0.152
< 5	37 (3.10)	45 (3.54)	53 (4.89)		22 (1.98)	27 (2.58)	31 (3.18)		24 (2.34)	22 (2.28)	35 (3.70)	
[5, 10)	301 (25.23)	292 (22.99)	248 (22.88)		205 (18.47)	234 (22.33)	209 (21.41)		196 (19.12)	198 (20.52)	203 (21.48)	
[10, 20)	496 (41.58)	561 (44.17)	467 (43.08)		479 (43.15)	453 (43.23)	449 (46.00)		460 (44.88)	411 (42.59)	420 (44.44)	
≥ 20	359 (30.09)	372 (29.29)	316 (29.15)		404 (36.40)	334 (31.87)	287 (29.41)		345 (33.66)	334 (34.61)	287 (30.37)	
Age at recruitment [n (%)]				0.112				0.892				0.977
< 35	1 041 (87.19)	1 094 (85.80)	965 (88.69)		973 (87.42)	915 (87.23)	850 (86.73)		895 (87.32)	844 (87.28)	832 (87.58)	
≥35	153 (12.81)	181 (14.20)	123 (11.31)		140 (12.58)	134 (12.77)	130 (13.27)		130 (12.68)	123 (12.72)	118 (12.42)	
Pre-pregnancy BMI [kg/m², n (%)]				0.014				0.741				0.570
< 18.5	129 (10.81)	132 (10.35)	131 (12.06)		119 (10.69)	128 (12.20)	109 (11.12)		126 (12.29)	102 (10.55)	114 (12.00)	
[18.5, 24.0)	808 (67.73)	867 (68.00)	769 (70.81)		772 (69.36)	699 (66.63)	685 (69.90)		679 (66.24)	673 (69.60)	656 (69.05)	
[24.0, 28.0)	182 (15.26)	220 (17.25)	144 (13.26)		167 (15.00)	171 (16.30)	143 (14.59)		171 (16.68)	143 (14.79)	140 (14.74)	
≥ 28.0	74 (6.20)	56 (4.39)	42 (3.87)		55 (4.94)	51 (4.86)	43 (4.39)		49 (4.78)	49 (5.07)	40 (4.21)	
Education level [years, n (%)]				0.111				0.003				< 0.001
< 12	213 (17.84)	262 (20.57)	191 (17.57)		154 (13.84)	180 (17.16)	189 (19.29)		134 (13.07)	173 (17.89)	189 (19.89)	
≥12	981 (82.16)	1 012 (79.43)	896 (82.43)		959 (86.16)	869 (82.84)	791 (80.71)		891 (86.93)	794 (82.11)	761 (80.11)	
Parity [n (%)]				0.164				0.316				0.540
Nulliparous	923 (77.30)	981 (76.94)	870 (79.96)		845 (75.92)	804 (76.79)	771 (78.67)		785 (76.66)	732 (75.85)	740 (77.98)	
Multiparous	271 (22.70)	294 (23.06)	218 (20.04)		268 (24.08)	243 (23.21)	209 (21.33)		239 (23.34)	233 (24.15)	209 (22.02)	
Smoking during pregnancy [n (%)]	3 (0.25)	6 (0.47)	6 (0.55)	0.511	3 (0.27)	4 (0.38)	3 (0.31)	0.896	1 (0.10)	2 (0.21)	4 (0.42)	0.328
Drinking during pregnancy [n (%)]	9 (0.75)	10 (0.78)	12 (1.10)	0.611	5 (0.45)	8 (0.76)	8 (0.82)	0.530	7 (0.68)	4 (0.41)	10 (1.05)	0.249

Supplementary Table 2 **Baseline characteristics by maternal depressive symptom tertiles during each trimester** (continued)

Characteristics	First trimester depression				Second trimester depression				Third trimester depression			
	Tertile 1 (n = 1 194)	Tertile 2 (n = 1 275)	Tertile 3 (n = 1 088)	*P*-value	Tertile 1 (n = 1 113)	Tertile 2 (n = 1 049)	Tertile 3 (n = 980)	*P*-value	Tertile 1 (n = 1 025)	Tertile 2 (n = 967)	Tertile 3 (n = 950)	*P*-value
Diabetes during pregnancy [n (%)]				0.366				0.105				0.194
No	848 (71.50)	899 (71.01)	800 (74.07)		804 (72.30)	760 (72.45)	696 (71.09)		729 (71.12)	683 (70.63)	711 (74.84)	
GDM	316 (26.64)	338 (26.70)	264 (24.44)		287 (25.81)	279 (26.60)	258 (26.35)		281 (27.41)	268 (27.71)	222 (23.37)	
DM	22 (1.85)	29 (2.29)	16 (1.48)		21 (1.89)	10 (0.95)	25 (2.55)		15 (1.46)	16 (1.65)	17 (1.79)	
GH [n (%)]	88 (7.45)	60 (4.75)	56 (5.20)	0.010	76 (6.84)	57 (5.44)	49 (5.01)	0.167	63 (6.15)	64 (6.62)	45 (4.74)	0.190
Mode of conception [n (%)]				0.331				< 0.001				< 0.001
ART	465 (38.94)	534 (41.88)	439 (40.35)		295 (26.50)	374 (35.65)	417 (42.55)		262 (25.56)	347 (35.88)	391 (41.16)	
Spontaneous	729 (61.06)	741 (58.12)	649 (59.65)		818 (73.50)	675 (64.35)	563 (57.45)		763 (74.44)	620 (64.12)	559 (58.84)	
Children												
Gestational weeks at delivery (weeks, mean ± SD)	39.53 ± 1.00	39.55 ± 0.99	39.53 ± 1.02	0.913	39.61 ± 1.01	39.55 ± 1.01	39.53 ± 0.97	0.129	39.61 ± 1.01	39.55 ± 1.01	39.55 ± 0.96	0.236
Infant sex [n (%)]				0.854				0.157				0.148
Male	639 (53.52)	668 (52.39)	577 (53.03)		579 (52.02)	532 (50.71)	538 (54.90)		537 (52.39)	478 (49.43)	511 (53.79)	
Female	555 (46.48)	607 (47.61)	511 (46.97)		534 (47.98)	517 (49.29)	442 (45.10)		488 (47.61)	489 (50.57)	439 (46.21)	
Birth weight (g, mean ± SD)	3 412.92 ± 412.01	3 427.23 ± 388.73	3 396.37 ± 412.73	0.181	3 417.70 ± 397.33	3 397.40 ± 414.13	3 410.35 ± 409.93	0.505	3 403.43 ± 401.07	3 396.95 ± 414.96	3 406.14 ± 403.06	0.878
SGA [n (%)]	45 (3.77)	32 (2.51)	38 (3.49)	0.178	33 (2.96)	46 (4.39)	33 (3.37)	0.186	41 (4.00)	35 (3.62)	33 (3.48)	0.813
LGA [n (%)]	178 (14.91)	188 (14.76)	154 (14.15)	0.866	160 (14.38)	148 (14.14)	137 (13.98)	0.966	133 (12.99)	140 (14.48)	130 (13.70)	0.627
Breastfeeding duration [months, n (%)]				0.795				0.088				0.060
< 6	201 (16.85)	211 (16.61)	172 (15.84)		201 (18.11)	175 (16.73)	142 (14.53)		182 (17.77)	143 (14.82)	134 (14.15)	
≥ 6	992 (83.15)	1 059 (83.39)	914 (84.16)		909 (81.89)	871 (83.27)	835 (85.47)		842 (82.23)	822 (85.18)	813 (85.85)	

P-values for categorical variables are based on Pearson's Chi-square test; for continuous variables, independent t-test was used for normally distributed data, and Wilcoxon rank-sum test was applied for non-normally distributed data.
Missing (n): Annual household income (10), parity (4), pre-pregnancy BMI (3), education level (2), smoking during pregnancy (4), drinking during pregnancy (3), diabetes during pregnancy (25), GH (35), LGA (2), SGA (2), breastfeeding duration (12).

Supplementary Table 3 **Comparison of baseline characteristics between excluded and included population in the final analysis**

Characteristics	Excluded population (n = 3 022)	Included population (n = 4 710)	P-value
Mothers			
Place of residence [n (%)]			<0.001
Rural	435 (14.39)	534 (11.34)	
Urban/Suburban	2 587 (85.61)	4 176 (88.66)	
Annual household income [10 000 CNY, n (%)]			< 0.001
< 5	176 (5.88)	164 (3.49)	
[5, 10)	719 (24.01)	1 049 (22.32)	
[10, 20)	1 222 (40.80)	2 030 (43.19)	
⩾20	878 (29.32)	1 457 (31.00)	
Age at recruitment [years, n (%)]			0.185
< 35	2 608 (86.30)	4 115 (87.37)	
⩾ 35	414 (13.70)	595 (12.63)	
Pre-pregnancy BMI [kg/m^2, n (%)]			0.101
< 18.5	401 (13.30)	557 (11.83)	
[18.5, 24.0)	2 070 (68.68)	3 224 (68.49)	
[24.0, 28.0)	425 (14.10)	712 (15.13)	
⩾ 28.0	118 (3.92)	214 (4.55)	
Education level [years, n (%)]			0.002
< 12	601 (19.95)	808 (17.16)	
⩾12	2 411 (80.05)	3 900 (82.84)	
Parity [n (%)]			0.001
Nulliparous	2 196 (72.84)	3 588 (76.24)	
Multiparous	819 (27.16)	1 118 (23.76)	
Smoking during pregnancy [n (%)]	23 (0.77)	19 (0.40)	0.051
Drinking during pregnancy [n (%)]	44 (1.47)	43 (0.91)	0.033
Diabetes during pregnancy [n (%)]			0.050
No	2 192 (74.74)	3 387 (72.29)	
GDM	690 (23.53)	1 219 (26.02)	
DM	51 (1.74)	79 (1.69)	
GH [n (%)]	167 (5.73)	249 (5.33)	0.490
Mode of conception [n (%)]			0.003
ART	908 (30.05)	1 570 (33.33)	
Spontaneous	2 114 (69.95)	3 140 (66.67)	
Children			
Gestational weeks at delivery (weeks, mean ± SD)	39.54 ± 1.02	39.58 ± 1.00	0.096
Infant sex [n (%)]			1.000
Male	1 591 (52.65)	2 480 (52.65)	
Female	1 431 (47.35)	2 230 (47.35)	
Birth weight (g, mean ± SD)	3 416.53 ± 406.95	3 409.90 ± 403.82	0.486
SGA [n (%)]	105 (3.59)	167 (3.55)	0.971
LGA [n (%)]	466 (15.94)	666 (14.15)	0.035
Breastfeeding duration [months, n (%)]			< 0.001
< 6	611 (22.75)	798 (16.99)	
⩾ 6	2 075 (77.25)	3 900 (83.01)	

P-values for categorical variables are based on Pearson's Chi-square test; for continuous variables, independent t-test was used for normally distributed data, and Wilcoxon rank-sum test was applied for non-normally distributed data.
Missing (n): Annual household income (10), parity (4), pre-pregnancy BMI (3), education level (2), smoking during pregnancy (4), drinking during pregnancy (3), diabetes during pregnancy (25), GH (35), LGA (2), SGA (2), breastfeeding duration (12).
Abbreviations: ART, assisted reproductive technology; BMI, body mass index; DM, diabetes mellitus; GDM, gestational diabetes mellitus; GH, gestational hypertension; LGA, large for gestational age; SD, standard deviation; SGA, small for gestational age.

Supplementary Table 4 **Distribution of psychological scores at different stages of pregnancy**

Pregnancy psychology (mean ± SD)	First trimester (n = 3 557)	Second trimester (n = 3 142)	Third trimester (n = 2 942)	P for trend
Anxiety score	38.74 ± 7.56	35.99 ± 6.83	36.82 ± 7.23	<0.001
Depression score	12.90 ± 7.86	8.72 ± 7.16	8.98 ± 7.50	<0.001
Stress score	12.67 ± 5.19	9.78 ± 5.82	9.43 ± 6.00	<0.001

The P for trend was calculated by including the pregnancy period coded as an ordinal variable in the regression model to assess the significance of trends across ordered groups.
Abbreviation: SD, standard deviation.

Supplementary Table 5 **Distribution of physical growth parameters in children**

Age (months)	1 (n = 3 745)	3 (n = 4 100)	6 (n = 4 030)	8 (n = 3 906)	12 (n = 4 488)	18 (n = 3 826)	24 (n = 3 678)	30 (n = 2 892)	36 (n = 4 059)	P for trend
Height/length for weight (mean ± SD)	−0.02 ± 1.04	0.48 ± 1.02	0.72 ± 1.02	0.70 ± 0.97	0.58 ± 0.97	0.42 ± 0.88	0.34 ± 0.90	0.29 ± 0.95	0.20 ± 1.03	<0.001
Overweight/obesity [n (%)]	96 (2.56)	266 (6.49)	393 (9.75)	326 (8.35)	306 (6.82)	145 (3.79)	129 (3.51)	128 (4.43)	177 (4.36)	<0.001

The P for trend was calculated by including the age variable, coded as an ordinal variable, in the regression model to assess the significance of trends across groups.
Abbreviation: SD, standard deviation.

Supplementary Table 6 **Associations between prenatal anxiety, stress, and children's overweight/obesity from 1 to 36 months of age**

Groups	Crude		Model 1		Model 2	
	OR (95% CI)	P-value	OR (95% CI)	P-value	OR (95% CI)	P-value
First trimester anxiety score						
Tertile 1	Reference		Reference		Reference	
Tertile 2	1.07 (0.86, 1.33)	0.528	1.05 (0.85, 1.30)	0.633	1.05 (0.85, 1.30)	0.638
Tertile 3	1.23 (0.99, 1.52)	0.059	1.22 (0.98, 1.51)	0.079	1.21 (0.98, 1.51)	0.083
P for trend		0.057		0.076		0.080
Second trimester anxiety score						
Tertile 1	Reference		Reference		Reference	
Tertile 2	0.89 (0.70, 1.12)	0.317	0.87 (0.68, 1.10)	0.239	0.87 (0.69, 1.10)	0.243
Tertile 3	1.19 (0.94, 1.51)	0.144	1.17 (0.92, 1.49)	0.206	1.16 (0.91, 1.48)	0.226
P for trend		0.157		0.217		0.236
Third trimester anxiety score						
Tertile 1	Reference		Reference		Reference	
Tertile 2	1.22 (0.95, 1.55)	0.112	1.23 (0.97, 1.57)	0.089	1.22 (0.96, 1.56)	0.103
Tertile 3	1.26 (0.97, 1.62)	0.084	1.26 (0.97, 1.65)	0.083	1.25 (0.96, 1.63)	0.100
P for trend		0.095		0.094		0.113
First trimester stress score						
Tertile 1	Reference		Reference		Reference	
Tertile 2	1.05 (0.84, 1.30)	0.663	1.06 (0.86, 1.32)	0.580	1.06 (0.85, 1.32)	0.600
Tertile 3	1.21 (0.98, 1.50)	0.077	1.22 (0.99, 1.52)	0.068	1.22 (0.98, 1.52)	0.069
P for trend		0.078		0.070		0.071
Second trimester stress score						
Tertile 1	Reference		Reference		Reference	
Tertile 2	1.08 (0.86, 1.36)	0.527	1.08 (0.86, 1.35)	0.510	1.07 (0.86, 1.35)	0.534
Tertile 3	1.12 (0.88, 1.42)	0.367	1.12 (0.88, 1.44)	0.350	1.12 (0.87, 1.43)	0.386
P for trend		0.360		0.342		0.377
Third trimester stress score						
Tertile 1	Reference		Reference		Reference	
Tertile 2	1.10 (0.86, 1.41)	0.456	1.12 (0.87, 1.43)	0.373	1.11 (0.87, 1.42)	0.404
Tertile 3	1.24 (0.97, 1.58)	0.086	1.23 (0.95, 1.57)	0.111	1.21 (0.94, 1.55)	0.130
P for trend		0.088		0.110		0.129

Model 1 was adjusted for annual household income, pre-pregnancy BMI, age at recruitment, education level, place of residence, and parity. Model 2 was adjusted based on Model 1, with the addition of breastfeeding duration. The associations were estimated using the generalized estimating equation (GEE) models. The P for trend was calculated by including the median maternal depression score within each tertile as a continuous variable in the regression model to assess the significance of trends across ordered groups.
Abbreviations: BMI, body mass index; CI, confidence interval; OR, odds ratio.

Supplementary Table 7 **Associations between prenatal depression and children's WLZ from 1 to 36 months of age**

Groups	Crude		Model 1		Model 2	
	β (95% CI)	*P*-value	*β* (95% CI)	*P*-value	*β* (95% CI)	*P*-value
First trimester depression score						
Tertile 1	Reference		Reference		Reference	
Tertile 2	0.03 (−0.03, 0.09)	0.330	0.03 (−0.03, 0.09)	0.404	0.03 (−0.03, 0.09)	0.405
Tertile 3	0.08 (0.01, 0.14)	0.022	0.09 (0.02, 0.15)	0.008	0.09 (0.02, 0.15)	0.008
P for trend		0.022		0.007		0.008
Second trimester depression score						
Tertile 1	Reference		Reference		Reference	
Tertile 2	0.01 (−0.06, 0.07)	0.807	0.01 (−0.06, 0.07)	0.853	0.01 (−0.06, 0.07)	0.877
Tertile 3	0.07 (0.00, 0.14)	0.040	0.07 (0.01, 0.14)	0.034	0.07 (0.00, 0.14)	0.041
P for trend		0.035		0.029		0.035
Third trimester depression score						
Tertile 1	Reference		Reference		Reference	
Tertile 2	0.00 (−0.07, 0.07)	0.998	0.00 (−0.07, 0.07)	0.985	0.00 (−0.07, 0.06)	0.950
Tertile 3	0.05 (−0.02, 0.11)	0.200	0.05 (−0.02, 0.12)	0.164	0.05 (−0.02, 0.11)	0.182
P for trend		0.181		0.145		0.161

Model 1 was adjusted for annual household income, pre-pregnancy BMI, age at recruitment, education level, place of residence, and parity. Model 2 was adjusted based on Model 1, with the addition of breastfeeding duration. The associations were estimated using the generalized estimating equation (GEE) models. The *P* for trend was calculated by including the median maternal depression score within each tertile as a continuous variable in the regression model to assess the significance of trends across ordered groups.
Abbreviations: BMI, body mass index; CI, confidence interval.

Supplementary Table 8 **Information for model selection in group-based trajectory modeling**

Number of trajectories	AIC	BIC	AvePP	Prop	OCC
1	98 159.07	98 192.89	1.00	1.00	—
2	88 912.70	88 988.80	0.95, 0.95	0.53, 0.47	18.54, 19.73
3	85 213.57	85 331.95	0.94, 0.91, 0.94	0.34, 0.46, 0.20	30.71, 12.08, 56.34
4	83 870.52	84 031.17	0.89, 0.86, 0.92, 0.93	0.41, 0.25, 0.14, 0.20	11.56, 17.64, 63.60, 48.73
**5**	**83 106.34**	**83 309.26**	**0.82, 0.85, 0.84, 0.92, 0.92**	**0.15, 0.27, 0.28, 0.13, 0.17**	**21.90, 15.08, 15.01, 73.90, 58.90**
6	82 561.03	82 806.23	0.82, 0.83, 0.84, 0.80, 0.90, 0.91	0.17, 0.24, 0.17, 0.22, 0.06, 0.15	19.98, 16.57, 27.31, 14.33, 132.29, 56.45

Bold font indicates the groups that were selected for this study based on consideration of all relevant indicators.
Abbreviations: AIC, Akaike Information Criterion; AvePP, Average Posterior Probability; BIC, Bayesian Information Criterion; OCC, Odds of Correct Classification; Prop, Proportion of Assignment.

Supplementary Table 9 **Distribution of children's physical development in different trajectory groups**

Age (months, mean ± SD)	Low-rising	High-stable	Moderate-stable	Very-high-stable	Low-stable
	(*n* = 701)	(*n* = 1 285)	(*n* = 1 328)	(*n* = 608)	(*n* = 788)
1	−0.65 ± 1.26	0.55 ± 0.66	−0.05 ± 0.63	0.82 ± 1.00	−0.83 ± 0.86
3	0.38 ± 1.00	0.94 ± 0.59	0.17 ± 0.57	1.75 ± 0.84	−0.68 ± 0.75
6	0.79 ± 0.95	1.13 ± 0.51	0.30 ± 0.51	2.17 ± 0.75	−0.49 ± 0.66
8	0.76 ± 0.83	1.08 ± 0.49	0.31 ± 0.50	2.13 ± 0.73	−0.48 ± 0.62
12	0.70 ± 0.84	0.95 ± 0.52	0.21 ± 0.51	1.92 ± 0.80	−0.55 ± 0.66
18	0.54 ± 0.77	0.77 ± 0.51	0.08 ± 0.50	1.60 ± 0.67	−0.61 ± 0.61
24	0.47 ± 0.80	0.68 ± 0.51	0.02 ± 0.51	1.54 ± 0.75	−0.69 ± 0.63
30	0.42 ± 0.91	0.63 ± 0.54	−0.10 ± 0.51	1.56 ± 0.90	−0.73 ± 0.68
36	0.34 ± 1.08	0.55 ± 0.62	−0.14 ± 0.56	1.47 ± 1.04	−0.87 ± 0.70

Abbreviation: SD, standard deviation.

Supplementary Table 10 Associations between prenatal depression and growth trajectories in children from 1 to 36 months

Groups	Very-high-stable *vs.* Moderate-stable		High-stable *vs.* Moderate-stable		Low-rising *vs.* Moderate-stable		Low-stable *vs.* Moderate-stable	
	OR (95% CI)	*P*-value	OR (95% CI)	*P*-value	OR (95% CI)	*P*-value	OR (95% CI)	*P*-value
First trimester depression score								
Tertile 1	Reference		Reference		Reference		Reference	
Tertile 2	1.09 (0.84, 1.43)	0.508	1.17 (0.94, 1.45)	0.151	0.91 (0.71, 1.17)	0.477	1.03 (0.81, 1.32)	0.820
Tertile 3	1.39 (1.06, 1.83)	0.019	1.33 (1.06, 1.66)	0.014	0.97 (0.75, 1.26)	0.826	1.12 (0.87, 1.45)	0.379
P for trend		0.020		0.014		0.785		0.388
Second trimester depression score								
Tertile 1	Reference		Reference		Reference		Reference	
Tertile 2	1.00 (0.74, 1.35)	0.987	0.93 (0.74, 1.17)	0.536	0.93 (0.71, 1.22)	0.590	0.93 (0.72, 1.20)	0.576
Tertile 3	1.39 (1.03, 1.86)	0.030	0.99 (0.78, 1.25)	0.918	1.01 (0.76, 1.33)	0.959	0.94 (0.72, 1.23)	0.637
P for trend		0.030		0.892		0.983		0.623
Third trimester depression score								
Tertile 1	Reference		Reference		Reference		Reference	
Tertile 2	1.07 (0.79, 1.47)	0.653	1.36 (1.07, 1.72)	0.011	1.08 (0.81, 1.44)	0.590	1.19 (0.91, 1.56)	0.212
Tertile 3	1.47 (1.09, 1.98)	0.012	1.24 (0.97, 1.58)	0.081	1.13 (0.85, 1.50)	0.399	1.15 (0.88, 1.52)	0.311
P for trend		0.011		0.073		0.391		0.293

The model was adjusted for annual household income, pre-pregnancy BMI, age at recruitment, education level, place of residence, and parity, breastfeeding duration. The associations were estimated using multinomial logistic regression models. The *P* for trend was calculated by including the median maternal depression score within each tertile as a continuous variable in the regression model to assess the significance of trends across ordered groups.
Abbreviations: BMI, body mass index; CI, confidence interval; OR, odds ratio.

Supplementary Table 11 Stratified analysis of the association between prenatal depression and children's overweight/obesity from 1 to 36 months of age, stratified by children's sex

Groups	Male (*n* = 2 480)		Female (*n* = 2 230)		*P* for interaction
	OR (95% CI)	*P*-value	OR (95% CI)	*P*-value	
First trimester depression score					0.824
Tertile 1	Reference		Reference		
Tertile 2	1.06 (0.80, 1.40)	0.709	0.85 (0.61, 1.20)	0.354	
Tertile 3	1.42 (1.08, 1.87)	0.012	1.19 (0.86, 1.66)	0.295	
P for trend		0.014		0.345	
Second trimester depression score					0.512
Tertile 1	Reference		Reference		
Tertile 2	0.94 (0.70, 1.28)	0.716	1.20 (0.82, 1.76)	0.358	
Tertile 3	1.19 (0.89, 1.58)	0.247	1.47 (0.98, 2.18)	0.061	
P for trend		0.221		0.060	
Third trimester depression score					0.070
Tertile 1	Reference		Reference		
Tertile 2	0.89 (0.66, 1.21)	0.448	1.45 (0.95, 2.21)	0.083	
Tertile 3	1.05 (0.78, 1.42)	0.750	1.56 (1.04, 2.35)	0.033	
P for trend		0.681		0.049	

The model was adjusted for annual household income, pre-pregnancy BMI, age at recruitment, education level, place of residence, and parity, breastfeeding duration. The associations were estimated using generalized estimating equation (GEE) models. The *P* for trend was calculated by including the median maternal depression score within each tertile as a continuous variable in the regression model to assess the significance of trends across ordered groups.
Abbreviations: BMI, body mass index; CI, confidence interval; OR, odds ratio.

Supplementary Table 12 **Stratified analysis of the association between prenatal depression and children's overweight/obesity from 1 to 36 months of age, stratified by mode of conception**

Groups	ART (n = 1 570)		Spontaneous (n = 3 140)		P for interaction
	OR (95% CI)	P-value	OR (95% CI)	P-value	
First trimester depression score					0.339
Tertile 1	Reference		Reference		
Tertile 2	0.99 (0.71, 1.37)	0.935	1.09 (0.82, 1.46)	0.561	
Tertile 3	1.23 (0.90, 1.69)	0.199	1.46 (1.10, 1.94)	0.008	
P for trend		0.205		0.007	
Second trimester depression score					0.064
Tertile 1	Reference		Reference		
Tertile 2	0.87 (0.59, 1.29)	0.494	1.35 (1.00, 1.82)	0.053	
Tertile 3	1.06 (0.73, 1.55)	0.743	1.41 (1.04, 1.90)	0.025	
P for trend		0.667		0.033	
Third trimester depression score					0.858
Tertile 1	Reference		Reference		
Tertile 2	1.22 (0.80, 1.87)	0.349	0.99 (0.71, 1.37)	0.941	
Tertile 3	1.34 (0.91, 1.98)	0.138	1.33 (1.00, 1.78)	0.053	
P for trend		0.140		0.042	

The model was adjusted for annual household income, pre-pregnancy BMI, age at recruitment, education level, place of residence, and parity, breastfeeding duration. The associations were estimated using the generalized estimating equation (GEE) models. The P for trend was calculated by including the median maternal depression score within each tertile as a continuous variable in the regression model to assess the significance of trends across ordered groups.
Abbreviations: BMI, body mass index; CI, confidence interval; OR, odds ratio.

Supplementary Table 13 **Stratified analysis of the association between prenatal depression and childhood overweight/obesity from 1 to 36 months, stratified by maternal pre-pregnancy BMI (kg/m²)**

Groups	BMI < 18.5 (n = 557)		18.5≤ BMI < 24.0 (n = 3 224)		24.0≤ BMI < 28.0 (n = 712)		BMI ≥ 28.0 (n = 214)		P for interaction
	OR (95% CI)	P-value	OR (95% CI)	P-value	OR (95% CI)	P-value	OR (95% CI)	P-value	
First trimester depression score									0.823
Tertile 1	Reference		Reference		Reference		Reference		
Tertile 2	1.40 (0.66, 2.97)	0.377	0.92 (0.70, 1.22)	0.565	1.27 (0.80, 2.02)	0.310	0.96 (0.48, 1.94)	0.915	
Tertile 3	1.52 (0.74, 3.14)	0.255	1.20 (0.93, 1.54)	0.168	1.54 (0.96, 2.48)	0.073	1.62 (0.76, 3.44)	0.212	
P for trend		0.250		0.191		0.076		0.206	
Second trimester depression score									0.755
Tertile 1	Reference		Reference		Reference		Reference		
Tertile 2	0.67 (0.30, 1.52)	0.337	1.14 (0.83, 1.57)	0.412	0.99 (0.60, 1.64)	0.984	1.46 (0.65, 3.29)	0.358	
Tertile 3	0.99 (0.44, 2.23)	0.975	1.50 (1.11, 2.02)	0.008	1.29 (0.79, 2.13)	0.310	1.02 (0.45, 2.32)	0.963	
P for trend		0.998		0.006		0.298		0.912	
Third trimester depression score									0.794
Tertile 1	Reference		Reference		Reference		Reference		
Tertile 2	1.18 (0.52, 2.68)	0.688	0.93 (0.67, 1.29)	0.660	1.16 (0.69, 1.97)	0.571	1.67 (0.73, 3.85)	0.227	
Tertile 3	1.65 (0.77, 3.51)	0.197	1.31 (0.97, 1.77)	0.082	1.36 (0.80, 2.30)	0.250	1.29 (0.56, 2.94)	0.549	
P for trend		0.194		0.047		0.251		0.569	

The model was adjusted for annual household income, age at recruitment, education level, place of residence, and parity, breastfeeding duration. The associations were estimated using the generalized estimating equation (GEE) models. The P for trend was calculated by including the median maternal depression score within each tertile as a continuous variable in the regression model to assess the significance of trends across ordered groups.
Abbreviations: BMI, body mass index; CI, confidence interval; OR, odds ratio.

Supplementary Table 14 **Sensitivity analysis of the association between prenatal depression and children's overweight/obesity from 1 to 36 months of age**

Groups	In AGA (n = 3 875)		Excluded diabetes during pregnancy (n = 3 387)		Excluded hypertensive disorders during pregnancy (n = 4 426)		Without missing covariate data (n = 4 679)	
	OR (95% CI)	*P*-value	OR (95% CI)	*P*-value	OR (95% CI)	*P* -value	OR (95% CI)	*P*-value
First trimester depression score								
Tertile 1	Reference		Reference		Reference		Reference	
Tertile 2	0.99 (0.76, 1.28)	0.934	1.01 (0.78, 1.32)	0.933	1.00 (0.80, 1.26)	0.994	1.02 (0.82, 1.26)	0.885
Tertile 3	1.28 (1.01, 1.63)	0.044	1.41 (1.11, 1.80)	0.005	1.29 (1.04, 1.60)	0.020	1.34 (1.08, 1.66)	0.008
P for trend		0.048		0.007		0.025		0.007
Second trimester depression score								
Tertile 1	Reference		Reference		Reference		Reference	
Tertile 2	1.05 (0.78, 1.41)	0.758	0.98 (0.74, 1.30)	0.899	1.09 (0.84, 1.41)	0.445	1.04 (0.81, 1.32)	0.547
Tertile 3	1.43 (1.08, 1.89)	0.014	1.37 (1.04, 1.81)	0.027	1.35 (1.06, 1.72)	0.017	1.35 (1.06, 1.70)	0.013
P for trend		0.011		0.021		0.015		0.010
Third trimester depression score								
Tertile 1	Reference		Reference		Reference		Reference	
Tertile 2	1.04 (0.78, 1.41)	0.722	1.00 (0.75, 1.34)	0.985	1.02 (0.79, 1.33)	0.874	0.99 (0.76, 1.28)	0.924
Tertile 3	1.40 (1.05, 1.87)	0.021	1.18 (0.89, 1.58)	0.249	1.19 (0.93, 1.53)	0.167	1.28 (1.01, 1.63)	0.040
P for trend		0.016		0.225		0.152		0.031

The model was adjusted for annual household income, pre-pregnancy BMI, age at recruitment, education level, place of residence, and parity, breastfeeding duration. The associations were estimated using the generalized estimating equation (GEE) models. The *P* for trend was calculated by including the median maternal depression score within each tertile as a continuous variable in the regression model to assess the significance of trends across ordered groups.
Abbreviations: BMI, body mass index; CI, confidence interval; OR, odds ratio.

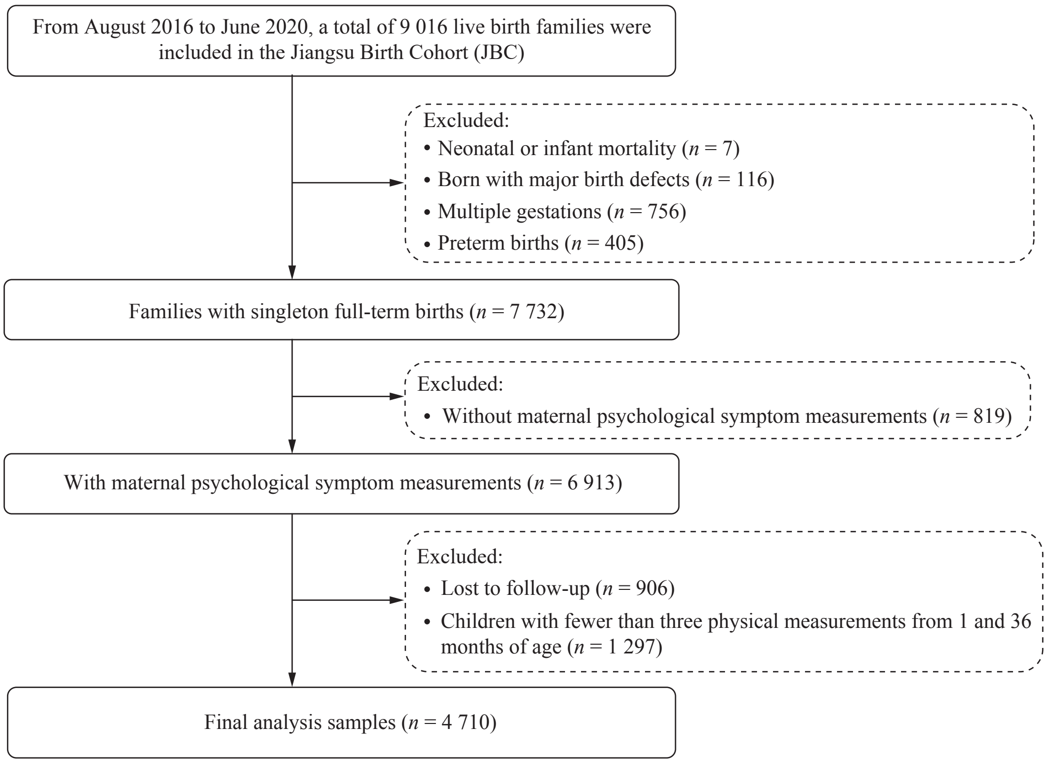

Supplementary Fig. 1 **Flowchart for the selection of study participants.**

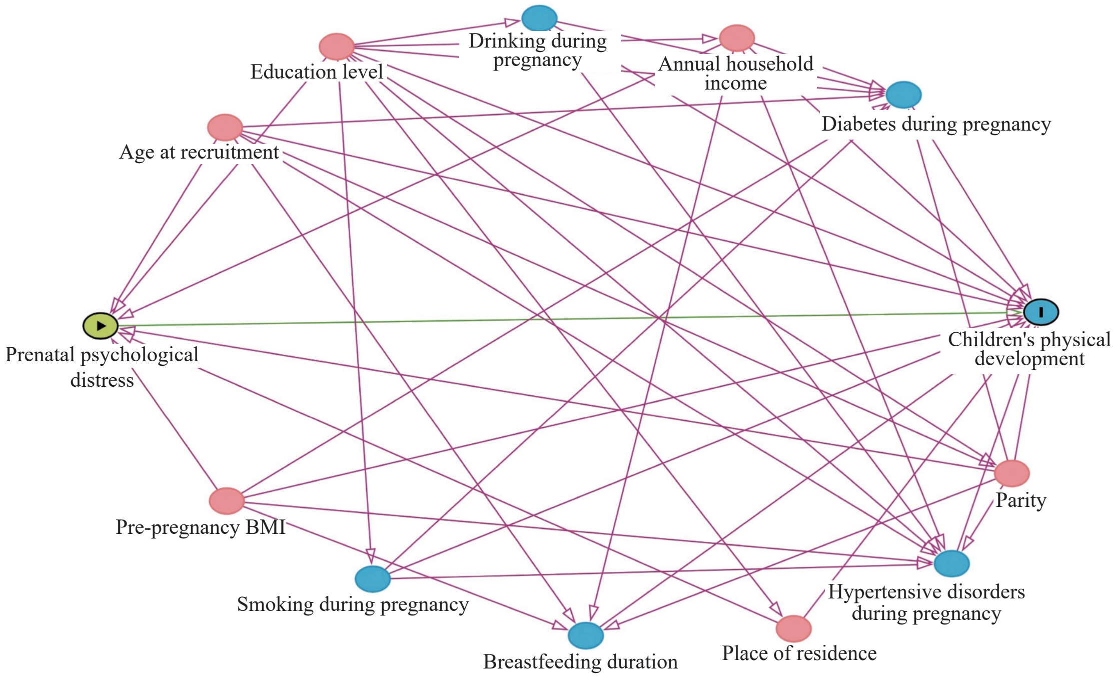

Supplementary Fig. 2 **Directed Acyclic Graph for potential confounders.** According to the DAG (Directed Acyclic Graph), the minimal sufficient adjustment sets for estimating the total effect of prenatal psychological distress on children's physical development include the following variables: annual household income, pre-pregnancy body mass index (BMI), age at recruitment, education level, place of residence, and parity.

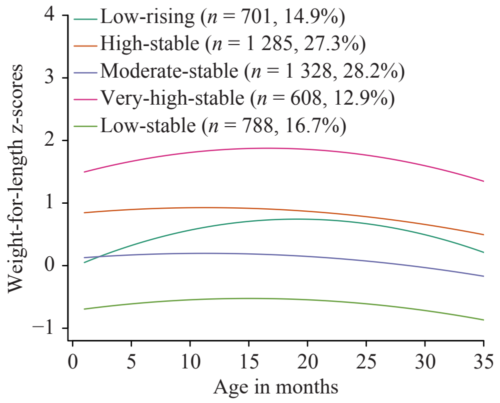

Supplementary Fig. 3 **Growth trajectories of weight-for-length z-scores (WLZ) from 1 to 36 months.** This figure displays five WLZ trajectory groups: very-high-stable, high-stable, moderate-stable, low-rising, and low-stable.
